# Portable SpectroChip-Based Immunoassay Platform for Rapid and Accurate Melamine Quantification in Urine Samples

**DOI:** 10.3390/toxics12120870

**Published:** 2024-11-29

**Authors:** Cheng-Hao Ko, Wei-Yi Kong, Abel Chernet Kabiso, Wei-Huai Chiu, Ashenafi Belihu Tadesse, Chitsung Hong, Chia-Fang Wu, Hung-Hsun Lin

**Affiliations:** 1 Graduate Institute of Automation and Control, National Taiwan University of Science and Technology, Taipei 106, Taiwan; d10712802@mail.ntust.edu.tw (W.-Y.K.); d11212804@mail.ntust.edu.tw (A.C.K.); mobetat41911@gmail.com (W.-H.C.); ashenafi.belihu@amu.edu.et (A.B.T.); 2 SpectroChip Inc., Hsinchu 310, Taiwan; cthong23@gmail.com; 3 Research Center for Environmental Changes, Academia Sinica (AS), Taipei 115, Taiwan; chiafangwu27@gmail.com; 4 Research Center for Environmental Medicine, Kaohsiung Medical University, Kaohsiung 807, Taiwan; katana1123@gmail.com; 5 Center of Teaching & Research, Kaohsiung Municipal Siaogang Hospital, Kaohsiung 812, Taiwan

**Keywords:** melamine detection, SpectroChip, spectral processing unit (SPU), point-of-care (POC), lateral flow immunoassay (LFIA), liquid chromatography–mass spectrometry (LC-MS)

## Abstract

Growing concerns about the health risks of melamine adulteration in food products highlight the urgent need for reliable detection methods. However, the long-term effects of chronic low-level melamine exposure remain inadequately explored. This study introduces THE ONE InstantCare platform, a portable immunoassay analyzer integrating a SpectroChip-based spectral processing unit (SPU) with lateral flow immunoassay (LFIA) for sensitive and accurate quantification of melamine in human urine. This platform provides a cost-effective, rapid, and user-friendly point-of-care (POC) solution for melamine detection. Analytical evaluations across eight melamine concentrations (0–100 parts per billion, ppb) achieved a limit of detection (LOD) of 1.91 ppb. Validation with 24 human urine samples demonstrated strong concordance with liquid chromatography–mass spectrometry (LC-MS), yielding an intraclass correlation coefficient (ICC) of 0.9220, a Pearson correlation coefficient of 0.9389, and 95% agreement in Bland–Altman analysis. High reproducibility was observed, with an intraday coefficient of variation (CV) of 6.53% and acceptable interday CV values, while interference studies confirmed reliability in the presence of common biological substances. By delivering results in approximately 10 min, THE ONE InstantCare platform significantly reduces analysis time compared to LC-MS, which typically requires several hours. This novel platform enhances food safety surveillance and advances human health risk assessments, particularly for evaluating melamine-linked kidney damage. Its versatility and robust performance make it a promising tool for environmental monitoring and clinical diagnostics, enabling the detection of diverse biomarkers with high sensitivity and reproducibility.

## 1. Introduction

Melamine is a trimer of cyanamide that forms a white crystalline molecule with the chemical formula C_3_H_6_N_6_ and skeleton 1,3,5-triazine. Products containing melamine are widely used in the manufacturing of plastics, glues, tableware, laminates, adhesives, and fertilizers [1]. The food industry has misused its high nitrogen content, illegally adding it to products such as milk to artificially inflate the protein content during food inspections. This fraudulent practice exploits the nitrogen-based methods used to assess protein levels, falsely classifying melamine-laced products as “protein-rich” [2,3]. Numerous public health emergencies and widespread recalls have been brought about by this adulteration, which has mostly affected dairy and pet food items. Large doses of melamine have the potential to combine with cyanuric acid to form crystals that are harmful to human health and insoluble in water. These crystals may cause serious health conditions, including kidney damage, bladder stones, and urinary tract obstructions [4,5,6,7]. Additionally, there is concern over the potential carcinogenic effects of melamine exposure [3,8].

In 2008, melamine-contaminated milk powder in China led to widespread kidney stones and renal failure in infants, resulting in 294,000 cases, 51,900 hospitalizations, and six deaths, with the contamination affecting 47 countries [9,10,11,12]. Melamine has an elimination halflife of a few hours, and is mostly (90%) eliminated unaltered in urine after ingestion [13,14]. According to experimental research, when combined with uric acid, the remaining amount (about 10%) results in the formation of massive insoluble crystalline formations or stones in the kidneys, ureters, and bladder [15]. According to in vitro evidence, ammeline, ammelide, and cyanuric acid are produced during alkaline hydrolysis of melamine [2]. In response to health concerns, particularly following the 2007–2008 melamine adulteration scandals involving milk and pet food, regulatory agencies such as the US Food and Drug Administration (FDA) and the European Food Safety Authority (EFSA) have set stringent safety standards for melamine levels in food products. These limits are based on scientific risk assessments of melamine exposure and toxicity, and were established to protect public health [16]. The FDA’s tolerance limits are set at 1 parts per million (ppm) for infant formula and 2.5 ppm for other milk products, both levels that global monitoring efforts have shown are typically not exceeded. These regulatory thresholds are designed to prevent the harmful effects of melamine while ensuring the safety and integrity of food products in international markets.

The consumption of trace amounts of melamine is generally considered to pose minimal health risk when levels remain well below the established safety limits [15]. However, recent studies have indicated that even low-level long-term exposure to melamine may result in adverse health outcomes. Research has shown that trace amounts of melamine can leach from melamine-based tableware when in contact with hot or acidic foods [17,18]. When ingested and absorbed through the gastrointestinal tract, melamine can combine with cyanuric acid, leading to the gradual formation of kidney stones. These stones can obstruct kidney tubules, eventually leading to kidney failure [19,20]. These results highlight the need for rapid and accurate detection techniques to identify and track traces of melamine, particularly to reduce long-term health hazards in susceptible groups such as newborns and young children. Current methods for detecting melamine include high-performance liquid chromatography (HPLC), gas chromatography–mass spectrometry (GC-MS), enzyme-linked immunosorbent assay (ELISA), near-infrared (NIR) spectroscopy, surface-enhanced Raman spectroscopy (SERS), liquid chromatography–tandem mass spectrometry (LC-MS/MS), and nuclear magnetic resonance (NMR). For trace amount detection, a few methods are particularly well suited, such as HPLC and LC-MS. The *LOD* for HPLC reaches 145 ppb, and for LC-MS it reaches as low as 0.66 ppb [21,22,23]. However, these methods are associated with high prices, require specialized equipment, and must be operated by trained personnel, making them largely confined to central clinical laboratories. Recent advancements have introduced more accessible and innovative methodologies, improving sensitivity and specificity in melamine detection. Li developed a surface-enhanced Raman scattering (SERS) method utilizing silver nanocube arrays, showing enhanced detection sensitivity for melamine in milk samples to 500 ppb [24]. Dikici introduced a “lab-on-pol” colorimetric sensor platform that uses melamine-imprinted membranes to detect melamine through color changes, achieving an *LOD* of 1248.6 ppb [25]. Guo et al. developed a highly sensitive and rapid method for detecting melamine in milk products by utilizing planar waveguide fluorescence immunosensors (PWFI), achieving an *LOD* of 6.6 ppb [26]. Similarly, Chen’s integration of DNA hydrogels with microfluidic chip technology demonstrated an *LOD* of 4.66 ppb [27]. However, further work is needed to validate these techniques in different conditions, such as in human urine, and make them more user-friendly.

A direct and effective method for assessing melamine exposure after consumption is the detection of melamine in urine, as it is primarily excreted unchanged [17]. There is significant need for a sensitive, specific, precise, and user-friendly test for melamine detection in urine to enhance public health monitoring and food safety. A POCT method for detecting melamine in urine or as a spot test for food items would be invaluable for controlling melamine adulteration and contamination. This rapid on-site detection would enhance patient safety and regulatory oversight, ensuring better protection against melamine exposure in both clinical and food safety settings [28,29,30,31]. The LFIA is a paper-based POCT method that is widely used because of its low cost, ease of use, and rapid format [32]. Traditional available LFIAs have poorer sensitivity and specificity compared to standard laboratory tests such as ELISA and polymerase chain reaction (PCR), limiting their impact in controlling disease. In recent works, improvement of these assays has included optimization of the assay kinetics and signal amplification using a reader system or by adding reagents [33]. Huang enhanced LFIA using magnetic nanoparticles, achieving an *LOD* of 400 ppb visible to the naked eye [34]. Qi utilized polyT-modified gold nanoparticles to reach an *LOD* of 2.52 ppb through spectrophotometric methods [35]. Yue further improved LFIA by employing fluorescent markers in conjunction with a fluorescence detection machine, resulting in an *LOD* of 27.37 ppb [36]. Zhong introduced an enhancement pad to LFIA, yielding an *LOD* of 10 ppb detectable by the naked eye [37]. Despite these advancements, the visual interpretation of LFIA results can be subjective, often leading to false negative reports in weakly positive samples. Consequently, LFIA-based rapid tests are primarily used for qualitative results. To address these limitations and improve the quantitative and sensitive capabilities of LFIA, especially for field or remote applications, we have developed a novel microspectrometer module and detection method, which we have integrated into the POCT reader known as THE ONE InstantCare.

A microspectrometer employs microelectromechanical system (MEMS) technology to consolidate the functions of a traditional spectrometer onto an optical chip, known as a SpectroChip. This technology enables the acquisition of continuous-spectrum and high-resolution reflectance spectra from the immunoassay test line region. A microspectrometer has been integrated into THE ONE InstantCare device, Spectrochip Inc., Hsinchu 310, Taiwan, which provides high-resolution results across a broad spectral range of 300 nm to 1100 nm [38]. This device has been employed in various POCT applications, including for quantification of interleukin-6 (IL-6) levels in serum to assess the severity of influenza infections in children. The system achieves high sensitivity, with a detection limit of 76.85 pg/mL. In a study involving 46 children, it showed strong correlation with ELISA (Rho 0.706, *p* < 0.001), with 78.3% sensitivity and 50.0% specificity for severe cases. Combining IL-6 with C-reactive protein (CRP) improved performance, reaching 85.7% sensitivity, 95.5% specificity, and an AUC of 0.911 [39]. For the colorimetric quantification of paraquat in urine, this device achieved a standard-curve R^2^ of 0.9989, a detection range of 0–100 ppm, and an *LOD* of 3.01 ppm. It demonstrated comparable performance to other methods, and was clinically validated with urine samples from paraquat-poisoned patients [40]. This device is also able to quantify neutralizing antibody levels in sera, demonstrating a strong correlation with the cPass ELISA assay (Pearson’s r = 0.864; ICC = 0.90138). This makes it affordable, user-friendly, and a promising alternative for assessing SARS-CoV-2 vaccine efficacy [41]. Furthermore, this device quantifies anti-SARS-CoV-2 immunoglobulin G (IgG) with high sensitivity, achieving a detection limit of 186 pg/mL, enabling early antibody detection in PCR-confirmed patients as early as day 3 after symptom onset [42]. All platforms have been validated using biological samples and compared against standard testing methods.

This study presents a novel approach for measuring melamine in urine by integrating THE ONE InstantCare device with lateral flow immunoassay (LFIA) technology. This method provides a cost-effective and sensitive solution for detecting and monitoring melamine levels in food products and point-of-care testing (POCT) settings. Furthermore, it enhances the quantitative sensitivity of the LFIA. By utilizing the high-resolution spectral analysis capabilities of THE ONE InstantCare device, the detection and quantification of melamine concentrations are improved, leading to greater precision and overall analytical performance. This advancement enhances the reliability of the assay and extends its applicability in clinical and food safety contexts. The current study represents a modified and expanded version of work presented at the conference [43].

## 2. Materials and Methods

### 2.1. Sample Preparation

Melamine samples were prepared using artificial urine spiked with specific concentrations of melamine: 0 ppb, 2.5 ppb, 5 ppb, 10 ppb, 25 ppb, 50 ppb, 75 ppb, and 100 ppb. The artificial urine used in this study was qUAntify Plus Control from BIO-RAD, Berkeley, CA, USA, which consists of two concentration levels: Level 1 and Level 2. To simulate real human urine, a 1:1 ratio of Level 1 to Level 2 was employed. For platform validation, an ex vivo study was conducted using 24 human urine samples collected from melamine manufacturing workers at Kaohsiung Medical University (KMU), Taiwan. The trial protocol received approval from the Institutional Review Board of Kaohsiung Medical University Chung-Ho Memorial Hospital (Approval Number: KMUHIRB-E(1)-20150281). These samples, collected in 2018, were analyzed for melamine content using both THE ONE InstantCare platform and LC-MS at KMU.

### 2.2. Lateral Flow Immunoassay (LFIA)

**Figure 1:**
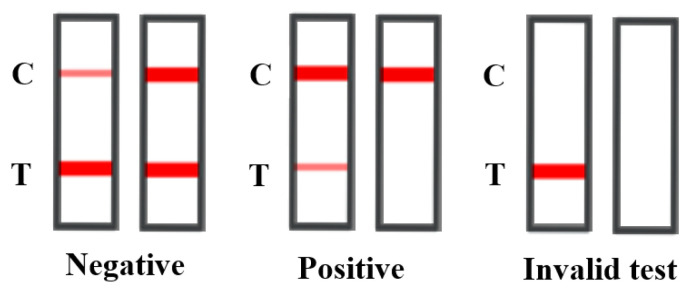
Interpretation of test strip results.

The LFIA selected for melamine detection was the Melamine (Mel) Rapid Test Kit produced by Vaccigen Biomedical Technology Co., Ltd., New Taipei City, Taiwan. This LFIA operates on the principle of competitive inhibition immunochromatography. A 200 µL sample interacts with a colloidal gold-labeled specific binding antibody as it moves along the strip. If melamine is present in the sample, it competes for antibody binding sites with the bovine serum albumin (BSA)-conjugated antigen on the nitrocellulose (NC) membrane’s test line (T-line), reducing the binding of the antibodies to the BSA antigen. This competition results in a visible color change on the T-line within 10 min. The control line (C-line) serves as a quality control indicator, confirming the test’s validity by displaying a colored band irrespective of the sample’s melamine content. As shown in Figure 1, if the color intensity of the T-line is equal to or darker than the C-line, this indicates that the melamine concentration in the sample is below 10 ppb; conversely, if the T-line appears significantly lighter than the C-line or is absent altogether, this signifies that the melamine concentration exceeds the *LOD* of 10 ppb.

### 2.3. THE ONE InstantCare Platform: SpectroChip–Spectrometer Platform

**Figure 2:**
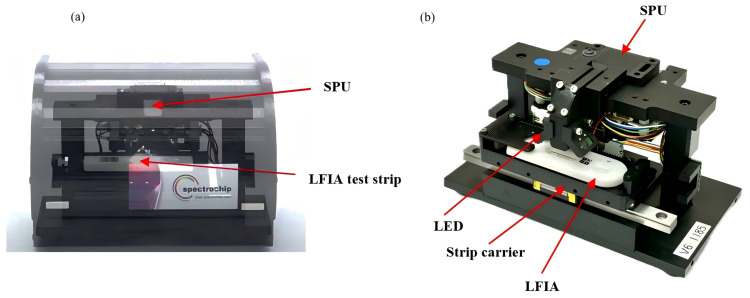
(**a**) THE ONE InstantCare platform and (**b**) inside view of the platform.

THE ONE InstantCare technology features a compact microspectrometer roughly the of the size matchbox that is equipped with a 1/3-inch 1.2 MP CMOS image sensor(AR0130, Onsemi, Phoenix AZ, USA) integrated into its spectral processing unit (SPU) module, as shown in Figure 2. Utilizing advanced SpectroChip technology, this device channels light into the SPU through an optical fiber, allowing the image sensor to capture the resulting spectrum, which is then transmitted via USB for data acquisition. This advanced system integrates a 12-bit analog-to-digital (A/D) converter to enhance light input precision, and employs an aberration-corrected concave micrograting to achieve flat-field focusing. As a result, the device exhibits a low overall noise level of 0.25%, which includes both sensor and optical noise, while effectively minimizing stray light interference. Operating within the 300–1000 nm spectral region and achieving a resolution of 5 nm, the device is capable of high-sensitivity detection at ppb levels. The detailed specifications of the SPU and system are presented in Table 1 and Table 2. THE ONE InstantCare system is particularly well suited for POC testing, seamlessly integrating with clinical diagnostic tools to deliver rapid results within 10 min for melamine detection. Its versatile design enhances performance and integration across a range of applications, including in the medical, optical, environmental, and industrial sectors.

### 2.4. Optical Pathway and Spectral Analysis of THE ONE InstantCare Platform

**Figure 3:**
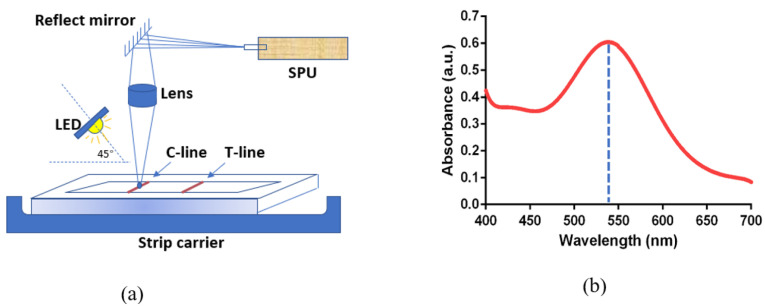
(**a**) Optical pathway of THE ONE InstantCare platform and (**b**) characteristic wavelength of colloidal gold.

A white-light LED positioned at a 45-degree angle relative to the optical axis serves as the light source for the system. The optical pathway is illustrated in Figure 3a, where it can be seen that the light emitted from the LED strikes the LFIA test strip and undergoes diffuse reflection from its surface. A strategically positioned lens focuses this reflected light onto the entrance of the SpectroChip’s. To maintain a compact design, a reflective mirror is utilized to redirect the light trajectory. The strip carrier allows for lateral movement, enabling the SpectroChip to sequentially scan both the C-line and T-line of the strip. The operating principle of this platform is based on measuring the absorbance of the C-line and T-line using the SPU. The appearance of a red line on the test strip indicates an antigen–antibody interaction in which gold nanoparticle antibody conjugates bind to antigens in the detection zone. When analyzed by a spectrometer, colloidal gold particles typically exhibit a spectral peak between 520 nm and 550 nm. Figure 3b presents the characteristic absorbance spectrum of the colloidal gold in the LFIA test for melamine detection.

Under the conditions of LED illumination, the SpectroChip collects the reflection spectra of the C-line ($S_{C}$) and T-line ($S_{T}$). The absorbance *A* of both lines can then be calculated using Equations (1) and (2):

(1) $$A_{C}=-\log_{10}\left(\frac{S_{C}}{S_{W}}\right)$$

(2) $$A_{T}=-\log_{10}\left(\frac{S_{T}}{S_{W}}\right)$$

where $S_{W}$ represents the reflection spectra of the white areas on the strip.

Based on the principle of competitive immunochromatography, we searched for the maximum values of $A_{C}$ and $A_{T}$ in the band range of 500–600 nm. To deduct the effect of the SPU baseline, the maximum absorbance was subtracted from the absorbance at 650 nm to obtain ΔA:

(3) $$\Delta A=A-A_{650\;nm}.$$

To assess the colorimetric difference between the C-line and T-line in the LFIA, the *Diff* variable was defined to capture the normalized difference in absorbance intensity:

(4) $$Diff=1-\frac{\Delta A_{T}}{\Delta A_{C}}$$

where $\Delta A_{T}$ and $\Delta A_{C}$ represent the absorbance intensities of the T-line and C-line, respectively. Higher *Diff* values indicate stronger analyte presence due to greater color contrast between the lines. This sensitive metric allows for accurate analyte quantification, distinguishing between positive and negative samples in complex matrices.

### 2.5. Urine Collection and Measurement by LC-MS

Midstream one-spot urine samples from the participants were collected following an overnight fasting period and stored at −80 °C for subsequent melamine analysis. The melamine concentration in the urine was quantified using an isotopic liquid chromatography–tandem mass spectrometry (LC-MS/MS) method, specifically employing the API 4000 Qtrap system (Applied Biosystems/MDS SCIEX, Concord, Toronto, ON, Canada) [44]. For melamine measurement, 1 mL of urine was processed using an Oasis MCX solid-phase extraction (SPE) cartridge (Waters, Milford, MA, USA). The eluate was evaporated under nitrogen gas and the resulting residue was reconstituted in 200 μL of mobile phase before undergoing LC-MS/MS analysis. This method achieved *LOD* of 0.8 ng/mL (ppb) for melamine in urine [45]. All measured values from the urine samples exceeded the *LOD*, ensuring that melamine concentrations were sufficiently detectable for analysis.

### 2.6. Experimental Procedure and Statistical Analysis

To determine the *LOD* and construct a standard curve, eight melamine standard solutions with concentrations ranging from 0 ppb to 100 ppb were prepared. The *LOD* is defined as the lowest analyte concentration that can be reliably distinguished from the limit of blank (*LOB*), marking the point at which detection becomes feasible. The *LOB* represents the highest apparent analyte concentration expected when replicates of a blank sample containing no analyte are tested [46]. The *LOD* was estimated as a 95% one-sided confidence limit by calculating *LOB* and the standard deviation (*SD*) of the blank according to Equations (5) and (6). Standard curves play a crucial role in quantitative analysis. They enable comparison of experimental data such as the absorbance intensity against known concentrations of a control standard, in turn facilitating the accurate determination of unknown sample concentrations. Each concentration was tested using LFIA strips in five replicates to ensure reliable data acquisition. The signal intensities from the C-line and T-line of each strip were recorded, then the difference in signal intensity between these lines was calculated to model the relationship with melamine concentration. The absorbance data corresponding to the C-line and T-line were further processed using a Savitzky–Golay (SG) filter to enhance signal clarity and reduce noise. This filtering technique allowed for more precise determination of the absorbance peak [47], which is critical for accurately assessing melamine concentrations. The *LOD* was identified as the lowest concentration of melamine that produced a signal distinguishable from the baseline (0 ppb) within a defined confidence interval. This approach ensured that even the smallest detectable concentration of melamine could be accurately quantified while maintaining a high level of statistical reliability.

(5) $$LOD={\mathrm{mean}}_{\mathrm{blank}\;\mathrm{sample}}+3\times {\mathrm{SD}}_{\mathrm{blank}}$$

(6) $$LOB={\mathrm{mean}}_{\mathrm{blank}}+1.645\times {\mathrm{SD}}_{\mathrm{blank}}$$

The reproducibility of the LFIA-based detection platform was rigorously evaluated in two experiments. In the first experiment, a melamine concentration of 10.0 ppb was tested 20 times in a single day, with measurements taken at different time points to assess intraday variability. In the second experiment, four melamine concentrations (including 0.0 ppb and 10.0 ppb) were tested in five replicates over three consecutive days to assess interday variability. For each experimental condition, the mean melamine concentration, *SD*, and *CV* were calculated. These metrics provided insights into the platform’s precision and consistency over time.

The specificity and potential interference of the melamine detection assay were evaluated through two additional sets of experiments. In the first experiment, melamine-free samples (0 ppb) were mixed with potential interfering substances commonly found in biological fluids, including aspirin (65 mg/dL), caffeine (50 mg/dL), human serum albumin (HSA, 600 mg/dL), and human bilirubin (40 mg/dL). Each interference condition was tested in triplicate and the resulting signal was compared to the baseline signal of 0 ppb melamine without interference. This analysis enabled us to determine whether any of the tested substances caused significant interference with the assay’s ability to accurately quantify melamine. In the second experiment, the same set of interfering substances was combined with a 10.0 ppb melamine standard solution, also tested in three replicates for each condition. Melamine levels were measured under each interference condition and paired *t*-tests were conducted to statistically compare the melamine concentrations with and without the presence of each interfering substance. If all *p*-values exceed the threshold of 0.05, this would indicate no significant interference with melamine detection in the urine samples.

To validate the platform against LC-MS, 24 human urine samples were analyzed using ICC, Bland–Altman analysis, and Pearson correlation coefficient. Reliability between the platform and LC-MS was assessed using ICC, where values below 0.5 were considered poor reliability, between 0.5 and 0.75 as moderate reliability, between 0.75 and 0.9 as good reliability, and above 0.9 as excellent reliability. Bland–Altman analysis was employed to evaluate the agreement between THE ONE InstantCare system and LC-MS, utilizing a 95% confidence interval to establish upper and lower limits. The correlation between the platform and LC-MS was assessed using Pearson correlation coefficients, with values between 0.25 and 0.5 indicating poor correlation, between 0.5 and 0.75 indicating moderate to good correlation, and between 0.75 and 1 indicating very good to excellent correlation. All data and related statistical analyses, including Pearson’s correlation coefficient, correlation analysis, and Bland–Altman analysis, were performed using GraphPad Prism software (version 6.01).

## 3. Results

### 3.1. Absorbance Spectra of the Rapid Test and Concentration Model

**Figure 4:**
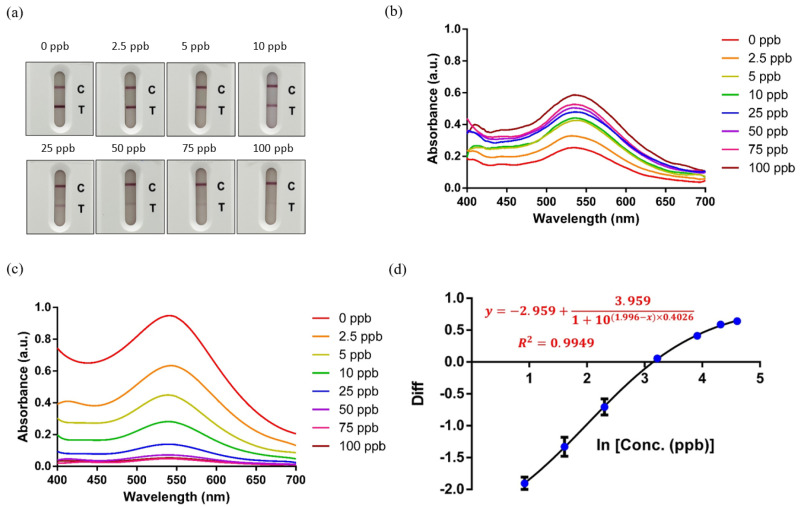
(**a**) Eight different concentrations of test strips, showing the absorbance curve of the C-line (**b**) and T-line (**c**) at different melamine concentrations and (**d**) the standard curve of melamine.

The absorbance corresponding to the C-line and T-line for the eight melamine concentration test strips is presented in Figure 4a. These test strips were analyzed using THE ONE InstantCare platform and the absorbance spectra for both the C-line and T-line were processed with the SG filter to reduce noise and enhance signal clarity, as depicted in Figure 4b,c. The maximum absorption peak was observed at around 550 nm. The presence of the C-line confirms the proper functioning of the test and ensures that the sample has adequately flowed through the strip. Because the LFIA system is based on competitive chromatographic immunoassay principles, the absorbance at 550 nm directly reflects the color intensity at the T-line, which is inversely correlated with the melamine concentration. At lower melamine concentrations, the absorbance is higher, producing a darker T-line color. This is because less melamine is bound to the antibody, permitting more antibody–antigen interaction on the strip. Conversely, at higher melamine concentrations the absorbance decreases, resulting in a lighter T-line color that may blend with the white background of the strip. This inverse relationship between absorbance and melamine concentration is key to the competitive inhibition mechanism of the assay, enabling precise determination of melamine levels.

For each tested sample, the difference in signal intensity between the T-line and C-line was calculated based on Equation (4). The melamine concentration for each sample was then logarithmically transformed to generate a linear relationship between signal response and concentration. To construct the concentration model, we applied the one-phase association equation to the data points corresponding to seven melamine concentrations, excluding the 0 ppb point, as logarithmic transformation does not apply to a concentration value of zero. This exclusion is common in immunoassay calibration, where baseline or blank signals are treated separately. The resulting standard curve shown in Figure 4d exhibits an excellent fit, with a coefficient of determination (R^2^) of 0.9949, demonstrating strong correlation between the predicted melamine concentration and the actual signal intensity differences measured across the test strips.

In the concentration–response model, the LOD is derived as the lowest melamine concentration that is reliably distinguishable from the baseline (0 ppb). The LOD was determined to be 1.91 ppb, calculated using a mean LOB of 1.53 ppb and SD of 0.13 ppb. These parameters are crucial for ensuring the robustness of the LOD determination, as they provide confidence that any signal exceeding 1.91 ppb can be reliably differentiated from background noise. This low LOD underscores the high sensitivity of THE ONE InstantCare platform for melamine detection, validating its effectiveness for detecting trace levels of melamine in complex biological matrices.

### 3.2. Reproducibility, Interference Testing, and Validation of Melamine Detection Using THE ONE InstantCare Platform

#### 3.2.1. Reproducibility Analysis

The results of the intraday reproducibility experiment conducted on the same day using test strips with a fixed melamine concentration are presented in Table S1a. The CV value for this experiment was 6.53%, classified as “excellent” (CV < 10%), indicating high precision and minimal variability in measurements under consistent conditions. For the interday reproducibility experiment performed over three consecutive days with four different melamine concentrations, the results are presented in Table S1b. As melamine concentration increases, the CV also increases, although all values remain within the “good” range (10% < CV < 20%). These findings demonstrate that the test strips maintained reliable performance and reproducibility across multiple days even at higher concentrations, confirming the robustness and accuracy of the platform for quantitative melamine detection under varying conditions.

#### 3.2.2. Interference Study

**Figure 5:**
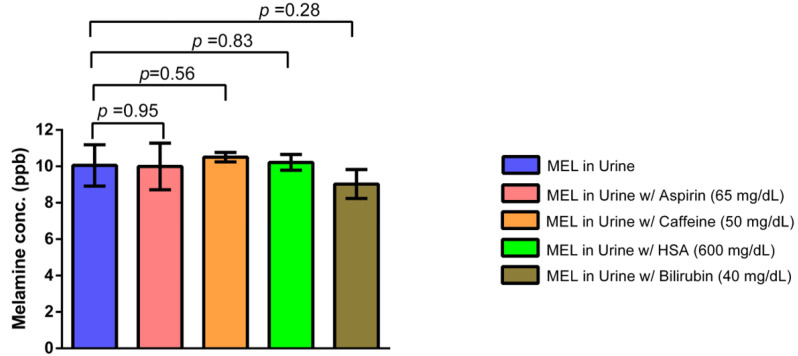
Comparison of melamine concentrations in urine with potential interfering substances.

After introducing potential interfering substances to the 0 ppb melamine solution, the results show that the measured melamine concentrations remained at or below the LOD across all interference conditions, as shown in Figure 5. Using paired *t*-tests revealed no significant differences in melamine concentration between the baseline and the urine samples containing these additives. Specifically, the *p*-values for melamine with aspirin (*p* = 0.95), caffeine (*p* = 0.56), HSA (*p* = 0.83), and bilirubin (*p* = 0.28) were all greater than 0.05, indicating no statistically significant change in melamine levels. Similarly, when interfering substances were added to the 10.0 ppb melamine solution, the observed mean melamine concentrations remained consistently close to 10.0 ppb. No significant differences were detected between the samples with and without interference, as shown in Table S2a,b. These results confirm that the assay’s performance was unaffected by the presence of common biological interferents, demonstrating the robustness and specificity of the detection platform for accurate melamine quantification in complex sample matrices.

#### 3.2.3. Validation with Human Urine Samples

**Figure 6:**
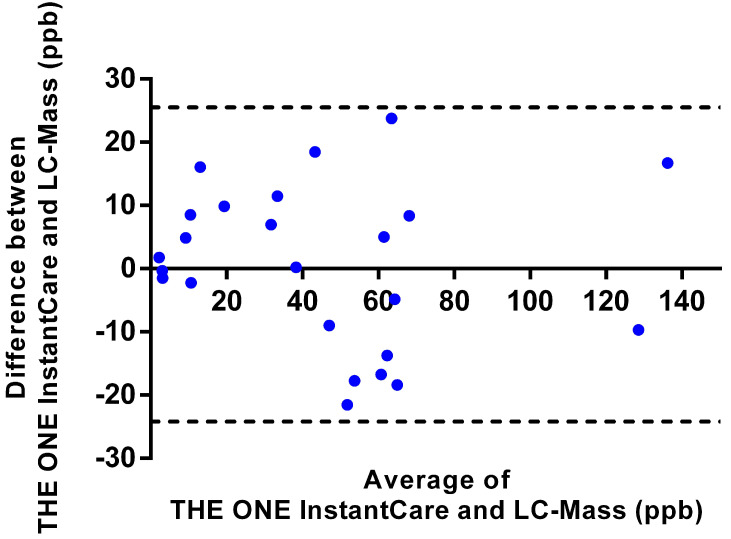
The results of Bland–Altman analysis comparing THE ONE InstantCare platform with LC-MS. The solid line represents the mean difference, and the dotted lines represent the 95% limits of agreement.

**Figure 7:**
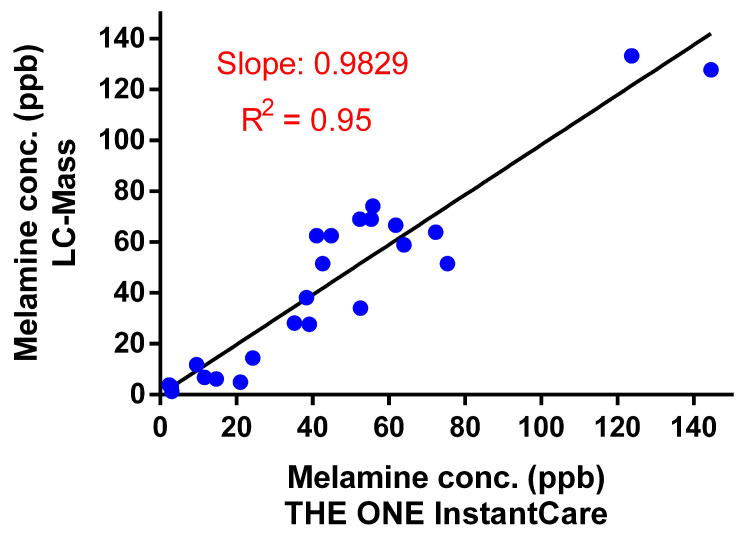
Results of correlation testing between THE ONE InstantCare platform and LC-MS. The scatter points represent individual data points, and the solid line indicates the line of best fit, showing the correlation between the two methods.

The validation study utilizing human urine samples demonstrated excellent agreement between THE ONE InstantCare platform and LC-MS measurements, with an intraclass correlation coefficient (ICC) of 0.9220, indicating high reliability. Bland–Altman analysis further corroborated this agreement, revealing that the differences between the ONE InstantCare platform and LC-MS measurements fell within the 95% confidence limits, specifically ranging from −25 ppb to +25 ppb, as illustrated in Figure 6. Additionally, the regression analysis presented in Figure 7 indicates a strong linear relationship between the two methods, with an R^2^ of 0.95, underscoring the platform’s accuracy in quantifying melamine concentrations. The calculated Pearson correlation coefficient of 0.9389 reinforces the high degree of correlation between the results from THE ONE InstantCare and LC-MS, confirming the platform’s robustness and suitability for melamine detection in biological samples.

## 4. Discussion

The melamine adulteration scandals in milk and pet food during 2007–2008 heightened global concerns about monitoring melamine levels in food products. Tolerable Daily Intake (TDI) standards for melamine have been established based on selected animal toxicity studies; however, instances of children developing melamine-related urolithiasis at exposure levels below the established TDI thresholds highlight significant public health concerns [8]. Moreover, chronic exposure to melamine has been associated with kidney damage, raising additional health concerns for both children and adults. The long-term health risks associated with low-level chronic melamine intake remain unclear. Furthermore, the potential health risks posed by hydrolytic derivatives of melamine such as cyanuric acid have received insufficient attention in food safety and environmental monitoring programs.

Several methods for detecting melamine are widely used due to their precision and sensitivity, including HPLC and LC-MS; however, these methods have significant limitations such as high costs, the need for advanced technologies, specialized reagents, and bulky equipment that is not portable. Additionally, their operation and maintenance require highly trained personnel, further increasing both complexity and expense. As a result, these methods are impractical for decentralized use and remain largely confined to central clinical laboratories, limiting their accessibility for rapid and widespread detection. For these reasons, the development of an effective platform for quantifying low levels of melamine and its derivatives such as the one proposed in this study is essential. The success of the utilized device in quantifying biomarkers such as IL-6, paraquat, and SARS-CoV-2 antibodies highlights its rapid, sensitive, and portable diagnostic capabilities. Similarly, an effective platform for detecting low levels of melamine is crucial for enhancing food safety and healthcare surveillance, and can enable timely interventions in both clinical and environmental monitoring.

In comparison to previous studies [10,22,24,25,27,32,34,35,36,37,48,49,50], the system developed in this study demonstrates superior sensitivity, achieving a limit of detection (LOD) for melamine as low as 1.91 ppb. When compared to LC-MS, which has a reported LOD of 0.8 ppb [44,45], both methods exhibit LOD values within the same order of magnitude. Validation with human urine samples revealed strong concordance between THE ONE InstantCare device and LC-MS, with an intraclass correlation coefficient (ICC) of 0.9220. Bland–Altman analysis further showed 95% agreement within a range of ±25 ppb. The reproducibility of THE ONE InstantCare device when integrated with lateral flow immunoassay (LFIA) was confirmed as well, with a coefficient of variation (CV) consistently classified as good or very good. Interference testing conducted with four substances at two melamine concentration levels (0 and 10 ppb) yielded consistent results, demonstrating compliance with US FDA regulations for in vitro diagnostic (IVD) devices. These findings underscore the robustness and reliability of the proposed platform for precise melamine quantification.

Table 3 compares various melamine detection methods, highlighting their preprocessing, LOD, read time, portability, and cost. THE ONE InstantCare platform stands out for its minimal preprocessing, 1.91 ppb LOD, read time of only 10 min, portability, and low cost. In contrast, despite their lower LODs, the HPLC, LC-MS, and GC-MS methods are non-portable, costly, and require extensive preprocessing. While SERS is portable and affordable, it is less sensitive, with a higher LOD of 500 ppb.

THE ONE InstantCare platform addresses the limitations of existing methods such as HPLC, GC-MS, and LC-MS, which despite their high precision require expensive equipment, specialized personnel, and lengthy analysis times. By integrating LFIA with a microspectrometer, this device ensures precise and quantitative measurements while remaining portable and user-friendly. Its compact dimensions (15 cm × 8 cm × 10 cm), lightweight design (490 g), and ability to operate with a standard smartphone charger make it highly practical for POCT settings where traditional laboratory testing is impractical. Requiring less than one hour of training, it offers affordability and efficiency, with additional features such as data storage and EHR integration that make it a robust solution for clinical, food safety, and environmental monitoring applications.

## 5. Conclusions

As global concerns about food safety continue to grow, effective monitoring of contaminants such as melamine has become increasingly important, particularly in light of past food scandals. In this study, a novel and highly sensitive POCT system that integrates THE ONE InstantCare device with LFIA technology is used for detecting melamine in human urine. With a remarkably low LOD of 1.91 ppb, this system offers superior sensitivity compared to existing methods, making it suitable for clinical and food safety applications. Detecting melamine is crucial in light of its association with kidney damage and urinary tract issues, particularly in vulnerable populations such as children and individuals with pre-existing kidney conditions. The proposed platform’s rapid detection capability supports early diagnosis and management of kidney-related diseases, significantly improving patient outcomes. Validation with human urine samples demonstrated strong correlation with LC-MS, confirming the platform’s high accuracy and reproducibility. In addition, it exhibited excellent intraday consistency and reliable performance across various concentrations, with interference studies indicating no effect from common biological substances. THE ONE InstantCare device offers substantial advantages over traditional methods, including reduced sample consumption, simplified operation, lower cost, and portability, making it ideal for use in non-laboratory settings. Overall, this novel approach addresses critical needs in food safety and public health by providing an accessible, reliable, and cost-effective tool for rapid melamine detection, especially in high-risk groups, potentially aiding in the prevention and management of kidney diseases associated with melamine exposure.

## Figures and Tables

**Table 1:** SPU parameter specifications.

Parameters	Specifications
Spectral Resolution	5 nm
Wavelength Range	300–1000 nm
Spectral Accuracy	±0.5 nm
Stray Light	0.04%
Spectral Dispersion Principle	Flat-field micro concave grating chip
Image Sensor	AR0130 CMOS
Spectral Pixel	1280 pixel
Optical Angle	24 degrees
A/D Conversion	12 bits
Analog Gain	1∼32
Dynamic Range	6000 (37.78 dB)
Dimensions (W × D × H)	44 mm × 34 mm × 8.7 mm

**Table 2:** THE ONE InstantCare platform parameter specifications.

Parameters	Specifications
Data Interface	mini-USB
Working Temperature	5~35 °C
Readout Time	2 min
Immunoassay	LFIA
Dimensions (W × D × H)	15 cm × 8 cm × 10 cm
Weight	490 g
Power Interface	micro-USB
Power Consumption	250 mW

**Table 3:** Comparison of detection methods and platforms.

Methods/Platform	Pre-Processing	LOD (ppb)	Read Time (min)	Portable	Cost
THE ONE InstantCare	Directly use	1.91	10	YES	LOW
HPLC	Extraction with buffer (HCOOH/NaCOOH); Centrifugation at 4 °C and treatment with ACN; centrifugation at 4 °C and treatment with CHCl_2_	145	60–120	NO	HIGH
LC-MS	Extraction from an Oasis MCX SPE cartridge; Dried with nitrogen	0.8	60–120	NO	HIGH
GC-MS	Extraction with ACN/H_2_O; Clean up with CARB/SCX cartridges; derivatization with BSTFA-TMCS	1–10	60–120	NO	HIGH
SERS	Directly use	500	<30	YES	LOW

## Data Availability

The data that support the findings of this study are available from the corresponding author upon reasonable request.

## Supplementary Materials

The following supporting information can be downloaded at: https://www.mdpi.com/article/10.3390/toxics12120870/s1, Table S1a: Testing results of a fixed concentration with 20 replicates performed on the same day; Table S1b: Various concentrations and three replicates performed on three consecutive days; Table S1c: Raw data of various concentrations and three replicates performed on three consecutive days; Table S2a: Interference study with 0 ppb melamine concentration; Table S2b: Interference study with 10.0 ppb melamine concentration; Table S3: The raw concentration data of LC-MS and The ONE InstantCare.
