# Supplementary material for: Portable SpectroChip-Based Immunoassay Platform for Rapid and Accurate Melamine Quantification in Urine Samples
Source: Toxics. 2024 Nov 29;12(12):870. doi: 10.3390/toxics12120870 (PMC11679044; doi:10.3390/toxics12120870)
Supplement: Supplementary file 1 [file toxics-12-00870-s001.zip › toxics-3322721-supplementary.pdf]

Supplementary Information

Table S1a: Testing results of a fixed concentration with 20 replicates performed on the same day

**10 ppb melamine in standard urine (ppb)**	
**20 replicates on the same day**	11.24
	11.94
	11.94
	11.94
	10.60
	10.02
	11.93
	11.24
	11.94
	11.24
	10.02
	10.60
	10.60
	10.02
	10.60
	10.60
	11.94
	11.94
	11.24
	10.60
**Mean**	**11.11**
**SD**	**0.73**
**CV**	**6.53%**

Table S1b: various concentrations and three replicates performed in three consecutive days

**Concentration (ppb)**	**Mean (ppb)**	**SD (ppb)**	**%CV**
5	5.06	0.30	5.89%
10	10.00	0.58	5.75%
25	27.19	2.16	7.95%
50	59.81	9.08	15.19%

Table S1c: Raw data of various concentrations and three replicates performed in three consecutive days

Raw data $Diff$				Raw data of concentration (ppb)			
5.06	10.00	27.19	59.81	5.06	10.00	27.19	59.81
-1.24	-0.74	0.08	0.56	5.53	9.56	26.67	68.80
-1.44	-0.77	0.17	0.59	4.39	9.28	30.74	76.48
-1.32	-0.77	0.17	0.54	5.07	9.23	30.59	65.39
-1.27	-0.63	0.05	0.47	5.33	10.76	25.38	55.07
-1.26	-0.62	0.12	0.50	5.43	10.95	28.46	59.69
-1.26	-0.82	0.11	0.45	5.39	8.80	27.80	52.28
-1.27	-0.69	0.04	0.48	5.38	10.08	25.27	56.18
-1.48	-0.66	0.04	0.50	4.22	10.48	25.16	59.29
-1.37	-0.63	0.03	0.38	4.79	10.85	24.61	45.08

Table S2a: Interference study with 0 ppb melamine concentration

Interference conditions	Negative urine (ppb)		
Aspirin (65 mg/dL)	< LOD	< LOD	< LOD
Caffeine (50 mg/dL)	< LOD	< LOD	< LOD
Human Serum albumin (HSA) (600 mg/dL)	< LOD	< LOD	< LOD
Bilirubin (40 mg/dL)	< LOD	< LOD	< LOD

Table S2b: Interference study with 10.0 ppb melamine concentration

Interference conditions	Positive urine (ppb)			Mean	Significant difference
Aspirin (65 mg/dL)	10.88	10.57	8.53	10.00	n.s
Caffeine (50 mg/dL)	10.57	10.22	10.73	10.51	n.s
Human Serum albumin (HSA) (600 mg/dL)	10.39	10.54	9.72	10.22	n.s
Bilirubin (40 mg/dL)	9.51	8.11	9.46	9.03	n.s

Table S3: The raw concentration of the LC-MS and The ONE InstantCare

ID	ΔA_C	ΔA_T	Diff.	The ONE InstantCare (ppb)	LC-mass (ppb)	Naked eye observer 1	Naked eye observer 2
#1	0.488	0.115	0.764	144.56	127.83	Positive	Positive
#2	0.451	0.122	0.7309	123.67	133.35	Positive	Positive
#3	0.416	0.177	0.575	72.28	63.92	Positive	Positive
#4	0.402	0.194	0.517	61.83	66.68	Positive	Positive
#5	0.397	0.209	0.475	55.77	74.13	Positive	Positive
#6	0.423	0.173	0.589	75.35	51.57	Positive	Positive
#7	0.385	0.257	0.331	41.06	62.60	Positive	Positive
#8	0.399	0.22	0.448	52.37	69.11	Positive	Positive
#9	0.371	0.365	0.016	24.29	14.43	Positive	Positive
#10	0.401	0.212	0.472	55.40	69.11	Positive	Positive
#11	0.383	0.288	0.249	35.22	28.23	Positive	Positive
#12	0.342	0.597	-0.745	9.51	11.76	Negative	Positive
#13	0.352	0.481	-0.366	14.70	6.17	Negative	Negative
#14	0.392	0.276	0.296	38.38	38.15	Positive	Positive
#15	0.391	0.271	0.306	39.11	27.64	Positive	Positive
#16	0.381	0.248	0.35	42.59	51.57	Positive	Positive
#17	0.404	0.19	0.53	63.97	58.94	Positive	Positive
#18	0.389	0.243	0.376	44.88	62.60	Positive	Positive
#19	0.398	0.219	0.449	52.56	34.08	Positive	Positive
#20	0.349	0.546	-0.564	11.65	6.76	Negative	Positive
#21	0.262	0.768	-1.930	2.38	3.86	Negative	Negative
#22	0.383	0.416	-0.085	21.05	4.97	Negative	Negative
#23	0.263	0.72	-1.738	3.08	1.33	Negative	Negative
#24	0.271	0.753	-1.781	2.91	3.24	Negative	Negative
